# Type II Gallbladder Perforation: A Management Conundrum or a Surgical Indecision?

**DOI:** 10.7759/cureus.95749

**Published:** 2025-10-30

**Authors:** Marco Youssef, Mina Aziz, Ashraf Rasheed

**Affiliations:** 1 Upper Gastrointestinal Surgery, The Grange University Hospital – Aneurin Bevan University Health Board NHS, Wales, GBR; 2 Surgery, The Grange University Hospital – Aneurin Bevan University Health Board NHS, Wales, GBR; 3 Hepatobiliary Surgery, Royal Gwent Hospital, Cardiff, GBR

**Keywords:** acute cholecystitis, cholecystostomy, gallbladder perforation, interventional radiology, niemeier type ii

## Abstract

Background: Gallbladder perforation (GBP) is a rare but serious complication of acute cholecystitis. Niemeier Type II, characterized by localized perforation with pericholecystic abscess, represents the most common subtype but lacks standardized management guidelines. This study aimed to evaluate clinical, radiologic, and comorbidity-related factors influencing treatment selection and outcomes in Type II GBP.

Methods: We retrospectively reviewed all radiologically diagnosed cases of GBP at a single university hospital between 2014 and 2022. Patients were classified according to Niemeier’s system, with Type II cases subdivided by management approach: cholecystectomy, percutaneous cholecystostomy, interventional radiology (IR) drainage, or antibiotics alone. Data collected included demographics, Charlson Comorbidity Index (CCI), CT findings, length of stay (LOS), reintervention, readmissions, and mortality. Multivariate analysis identified predictors of management strategy and outcomes.

Results: Ninety-one patients were identified, of whom Type II perforation accounted for 72 cases. Management strategies included cholecystectomy in 9 (13%) , cholecystostomy in 12 (17%), IR drainage in 17 (24%), and antibiotics alone in 33 (46%). One patient underwent combined IR drainage and cholecystostomy. Patients undergoing surgery had the lowest CCI, while conservative strategies were more common among older patients with a higher comorbidity burden. Gallbladder distension predicted cholecystostomy, while loculated collections were associated with IR drainage. Receiver operating characteristic (ROC) analysis demonstrated that a CCI greater than 5.5 effectively excluded patients from cholecystectomy. No significant differences were observed in mortality, LOS, or reintervention rates between treatment groups.

Conclusion: Comorbidity burden, age, and CT morphology are key determinants of management in Type II GBP. While surgery remains the definitive treatment, individualized nonoperative strategies can achieve comparable short- and intermediate-term outcomes in high-risk patients. Larger multicenter studies are required to establish evidence-based guidelines.

## Introduction

Gallbladder perforation (GBP) complicates 2-10% of acute cholecystitis cases and is frequently associated with comorbidities such as diabetes, hypertension, and cardiovascular disease [1-3]. The fundus is the most common site of perforation, reflecting its relatively poor vascular supply, which is further compromised by distension in unresolved cholecystitis [4].

Niemeier’s 1934 classification describes three types of GBP: Type I, chronic cholecystoenteric fistula; Type II, subacute localized perforation with pericholecystic abscess; and Type III, acute free perforation with generalized peritonitis [5]. Type II is the most commonly reported subtype in published series [2,6].

Management varies by type: Type III usually requires emergency surgery, whereas Type I may be approached electively. The optimal strategy for Type II, however, remains controversial, with debate between conservative and surgical approaches [2,6,7]. This study reports a nine-year experience of Type II GBP management at a university hospital in South Wales, United Kingdom.

## Materials and methods

Patient selection and data collection

We retrospectively reviewed all radiologically diagnosed cases of spontaneous GBP between 2014 and 2022. Type II perforation was defined radiologically by the presence of a focal wall defect, localized abscess, or contained bile leak, verified by consultant radiologists. Cases were classified according to Niemeier’s system. Data collected included demographics, perforation type, radiologic findings, Charlson Comorbidity Index (CCI), management approach, length of stay (LOS), prior admissions, subsequent interventions or readmissions, and mortality.

Type II GBP was further subdivided by management into four groups: cholecystectomy, percutaneous cholecystostomy, interventional radiology (IR) drainage, and antibiotics alone. LOS was measured from the date of intervention, and mortality was defined as in-hospital death. Intermediate follow-up was defined as 3-12 months post-discharge [8], focusing on readmissions and reinterventions.

Statistical analysis

Statistical analysis was performed using IBM SPSS Statistics for Windows, Version 27 (Released 2020; IBM Corp., Armonk, New York). Continuous variables were expressed as mean (± standard deviation) when normally distributed and as median (interquartile range, IQR) when not normally distributed, while categorical variables were presented as number (percentage). Group comparisons were performed using one-way ANOVA for normally distributed continuous variables and the chi-square test for categorical variables. Multivariate analysis was applied to identify predictors of outcomes, with statistical significance set at p < 0.05.

## Results

Between 2014 and 2022, 2,430 cases of cholecystitis were diagnosed, of which 91 (3.7%) involved GBP. The mean age was 70.8 ± 13.2 years, and 51 (56%) were male. Cholelithiasis was present in 80 (88%) cases. According to Niemeier’s classification, 72 (79%) had Type II (localized), 11 (12%) had Type I (chronic fistulation), and 8 (9%) had Type III (generalized peritonitis). The overall median length of stay was eight days (interquartile range, 6-13.5), with an overall mortality of 11 (12%). Age and sex distribution did not differ significantly among the three perforation groups (Table 1).

**Table 1:** Demographics across Gallbladder Perforation Types

Parameter	Total	Type III	Type II	Type I
Number of cases	91	8 (9%)	72 (79%)	11 (12%)
Age (mean)	70.76	69.4	70.5	73.3
Sex (M/F)	51/40	7/1	40/32	4/7

The mean number of comorbidities was 3.98 ± 2.08, with hypertension (42/91, 46%), diabetes (38/91, 42%), heart disease (28/91, 31%), and COPD (10/91, 11%) being the most common. CCI scores showed no significant variation across groups (ANOVA p = 0.32), and similarly, the mean number of comorbidities did not differ significantly (ANOVA p ≈ 0.22) (Table 2).

**Table 2:** Comorbidities and Charlson Comorbidity Index Across Gallbladder Perforation Types

Parameter	Total	Type III Acute	Type II Subacute	Type I Chronic	P-value
Diabetes	38/91 (41.8%)	2/8	32/72	4/11	0.53
Hypertension	42/91 (46.2%)	3/8	34/72	5/11	0.87
Heart disease	28/91 (30.7%)	3/8	23/72	2/11	0.60
Chronic obstructive pulmonary disease (COPD)	10/91 (10.9%)	1/8	8/72	0/11	0.50
Mean Charlson Comorbidity Index (CCI)	4.86 ± 2.67	4.13 ± 2.64	5.00 ± 2.78	4.45 ± 1.86	0.32
Average number of comorbidities	3.98 ± 2.09	3.38 ± 1.63	4.2 ± 2.04	2.82 ± 2.40	0.22

Length of stay and mortality were not statistically different between groups. The longer hospital stay in Type I likely reflects the chronicity and technical complexity of the fistulating disease. Reintervention/readmission occurred in 1/8 (12.5%) Type III, 16/72 (22.2%) Type II, and 6/11 (54.5%) Type I cases, with overall significance (χ² p = 0.049) driven by higher rates in Type I versus Type II (Fisher’s exact p = 0.046) (Table 3).

**Table 3:** Summary of Clinical Outcomes across Gallbladder Perforation Types LOS: length of stay, IQR: interquartile range.

Variables	Total	Type III	Type II	Type I	p-value
LOS	8 days (IQR 6–13.5)	10.5 days (IQR 6.75–14.5)	8 days (IQR 6–13.5)	9 days (IQR 6–16)	0.815
Re-intervention/admission	23/91	1/8	16/72	6/11	0.049
Mortality	11/91	1/8	10/72	0/11	0.42

Type II perforation

Type II perforation accounted for 72 cases (40 male, 32 female). Management strategies included cholecystectomy in 9 (13%) (2 open, 3 laparoscopic converted to open, and 4 laparoscopic), cholecystostomy in 12 (17%), IR drainage in 17 (24%), and antibiotics alone in 33 (46%). One patient underwent combined IR drainage and cholecystostomy.

Demographics and comorbidities in Type II perforations

We found no statistically significant difference in age or number of comorbidities among the four management groups (ANOVA p = 0.53 and p = 0.135, respectively). Sex distribution differed significantly among management groups (χ² p = 0.038), with a higher proportion of males in the cholecystectomy and IR drainage groups and more females in the IV antibiotics group.

The mean CCI was 5.21 ± 2.47 for the group treated with antibiotics, 4.47 ± 2.15 for the IR drainage group, 5.66 ± 3.14 for the cholecystostomy group, and 2.77 ± 1.98 for the cholecystectomy group. One-way ANOVA revealed a significant difference in CCI across the four management groups (F = 3.146, p = 0.031). Post hoc analysis showed that the cholecystostomy group had a significantly higher mean CCI (mean = 5.66) than the cholecystectomy group (mean = 2.77) (p = 0.023). A summary of demographics and CCI is shown in Table 4.

**Table 4:** Demographics and Charlson Comorbidity Index (CCI) in Different Management Types

Characteristic	Cholecystectomy	Cholecystostomy	IR Drainage	IV Abxs	p-value
Sex (M/F)	9 (7/2)	12 (6/6)	17 (13/4)	33 (13/20)	0.038
Mean age	62.3	71.33	65.4	74.5	0.53
CCI (mean ± SD)	2.77 ± 1.98	5.66 ± 3.14	4.47 ± 2.15	5.21 ± 2.47	0.031

CT findings in type II perforation

On CT, loculated collection was the most frequent finding, seen in 27/72 (38%) cases and most often managed with IR drainage in 13/27 (48%). Pericholecystic collection was present in 15/72 (21%) cases, of which 11/15 (73%) were treated with IV antibiotics.

A gallbladder wall defect was identified in 49/72 (68%) cases, most commonly at the body in 21/49 (43%) and at the fundus in 18/49 (37%). Gallbladder distension, defined as diameter >4 cm [9], was observed in 13/72 (18%) patients, predominantly in the cholecystostomy group (10/12, 83%).

CT morphology was significantly associated with management type (Pearson χ² p = 0.048; Fisher-Freeman-Halton p = 0.016). Details of CT findings are summarized in Tables 5-8.

**Table 5:** Site of Perforation in Different Management Types

Site of Perforation	Cholecystectomy (n=9)	Cholecystostomy (n=12)	IR Drainage (n=17)	IV Abxs (n=33)	Cholecystostomy + IR (n=1)	Total
Fundus	2	3	4	9		18
Body	2	5	4	10		21
Post into liver	1	2	2	2		7
Neck	1	0	0	2		3
Unknown	3	2	7	10	1	23

**Table 6:** CT Findings (Collection) by Management Type

CT Findings	Cholecystectomy	Cholecystostomy	IR Drainage	IV Abxs	Total
Loculated collection	3	3	13	8	27
Subcapsular collection	2	1	2	0	5
Pericholecystic collection	3	2	0	10	15
No or minimal collection	1	3	1	9	14
Extensive	0	1	0	0	1
Liver	0	2	1	6	9
Total	9	12	17	33	71

**Table 7:** Defect on CT by Management Type

Defect on CT	Cholecystectomy	Cholecystostomy	IR Drainage	IV Abxs	Total
Not identified	3	2	7	10	22
Defect	6	10	10	23	49
Total	9	12	17	33	71

**Table 8:** GB Distension on CT by Management Type GB: gallbladder.

Variable	Cholecystectomy	Cholecystostomy	IR Drainage	IV Abxs	Total
GB distension	3/9	10/12	2/17	11/33	36/72

Outcome and follow-up

Among patients with Type II perforation (n = 71), the median hospital stay did not differ significantly between management groups (Kruskal-Wallis p = 0.261). The median LOS was 8 days (IQR 7-11) for cholecystectomy, 7.5 days (IQR 6-13.3) for cholecystostomy, 12 days (IQR 9-18) for IR drainage, and 8 days (IQR 5-13) for IV antibiotics. Although numerically longer in the IR drainage group, this difference was not statistically significant.

At intermediate follow-up (3-12 months post-discharge), readmissions occurred in 3/9 (33%) cholecystectomy patients (bile leak requiring ERCP, drain review, and suspected duodenal perforation managed conservatively), 4/12 (33%) in the cholecystostomy group (including one recurrent Type II perforation), 3/17 (18%) after IR drainage, and 6/33 (18%) following IV antibiotics (due to cholecystitis, cholangitis, jaundice, or ERCP-related issues). No significant difference was observed across groups (Fisher’s exact p = 0.549).

Inpatient mortality was 0/9 (0%) in the cholecystectomy group, 3/12 (25%) following cholecystostomy (mean age 85.3 years, mean CCI 6.3), 3/17 (18%) after IR drainage (patients with advanced malignancy, cirrhosis, or multiple comorbidities), and 4/33 (12%) in the IV antibiotics group (mean age 80.3 years, mean CCI 7). Mortality did not differ significantly between groups (p = 0.432). A summary of outcomes is illustrated in Table 9.

**Table 9:** Outcome in Different Management Types

Outcome	Cholecystectomy	Cholecystostomy	IR Drainage	IV Abxs	p-value
Hospital stay median (days)	8 days (IQR 7–11)	7.5 days (IQR 6–13.25)	12 days (IQR 9–18)	8 days (IQR 5–13).	0.261
Mortality (n/N)	0/9	3/12	4/17	6/33	0.432
Further admission/intervention (n/N)	3/9	3/12	3/17	6/33	0.549

Interval cholecystectomy was undertaken following stabilization of the acute illness. This was performed in 6/33 (18.2%) patients managed initially with IV antibiotics, 4/17 (23.5%) after IR drainage, and 6/12 (50%) following cholecystostomy. Reasons for non-performance included death in the community, patient refusal, ongoing wait at the time of analysis, and medical unfitness due to frailty or comorbidities.

Multivariate analysis in Type II perforation

Multivariate analysis was undertaken to assess clinical and radiological predictors of management strategy in Type II perforation. Multinomial logistic regression showed that both CCI and age were significant determinants of management. Compared with cholecystectomy, higher CCI increased the likelihood of cholecystostomy (OR 3.80, p = 0.016) and IV antibiotics (OR 2.76, p = 0.048), with a similar trend for IR drainage (OR 2.57, p = 0.072). Older age also favored conservative approaches, being associated with higher odds of IV antibiotics (OR 0.889, p = 0.017) and cholecystostomy (OR 0.852, p = 0.027). CT findings, when grouped, were significant overall predictors (p < 0.001).

**Figure 1:**
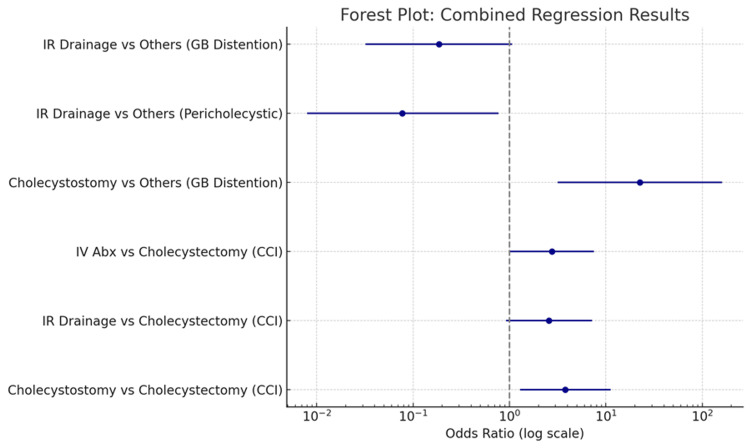
Forest Plot: Combined Regression Results GB: gallbladder, CCI: Charlson Comorbidity Index.

Binary logistic regression further explored the effect of CT features. Gallbladder distension independently predicted cholecystostomy (OR 22.6, p = 0.002). Pericholecystic collection was negatively associated with IR drainage (OR 0.077, p = 0.029), while gallbladder distension showed a borderline negative association (OR 0.186, p = 0.059). Loculated collections demonstrated a non-significant positive trend toward IR drainage (OR ≈ 2.1, p ≈ 0.12). Collectively, these results indicate that comorbidity burden, patient age, and specific CT features strongly influence the choice of management. Full regression outputs are summarized in Table 10, with a forest plot of key findings presented in Figure 1. 

**Table 10:** Key Predictors of Management Strategy in Type II Gallbladder (GB) Perforation (Multivariate Analysis) CCI: Charlson Comorbidity Index.

Comparison	Predictor	Key Effect	p-value
IV Abx vs Cholecystectomy	Age	↑ likelihood of IV Abx	0.017
IV Abx vs Cholecystectomy	CCI	↑ likelihood of IV Abx	0.048
Cholecystostomy vs Cholecystectomy	Age	↑ likelihood of cholecystostomy	0.027
Cholecystostomy vs Cholecystectomy	CCI	↑ likelihood of cholecystostomy	0.016
Cholecystostomy vs Cholecystectomy	GB distension	Strong predictor of cholecystostomy	0.002
IR Drainage vs Others	Pericholecystic collection	↓ likelihood of IR drainage	0.029

ROC analysis

**Figure 2:**
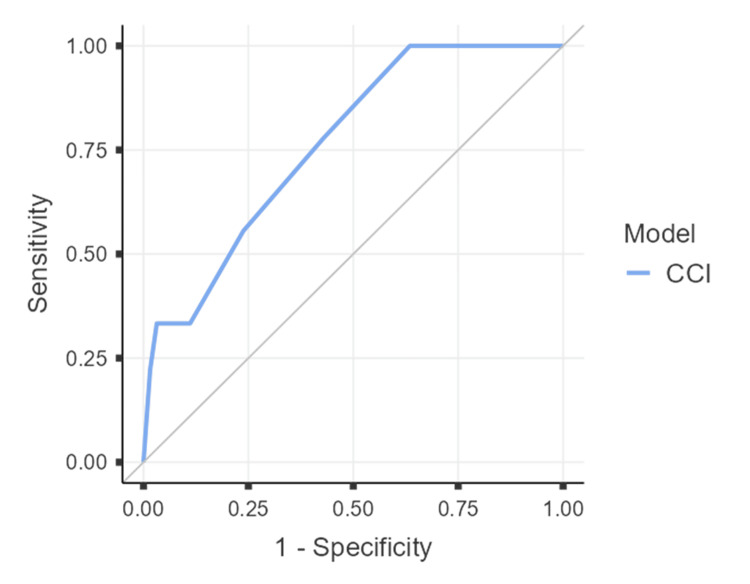
Receiver Operating Characteristic Curve CCI: Charlson Comorbidity Index.

ROC analysis assessed the ability of CCI to predict selection of cholecystectomy versus conservative management. The area under the curve (AUC) was 0.765 (95% CI 0.614-0.915, p < 0.001), indicating fair discrimination. An optimal cut-off of >5.5 was identified by Youden’s Index, yielding a sensitivity of 100% (95% CI 66.4-100%) and a specificity of 36.5% (95% CI 24.7-49.6%). The negative predictive value was 100%, though the positive predictive value was limited (18.4%). Thus, higher CCI strongly predicted avoidance of cholecystectomy but lacked specificity for identifying surgical candidates. Results are shown in Figure 2 and Table 11.

**Table 11:** Receiver Operating Characteristic Curve Summary AUC: area under the curve.

	AUC	Std. Error	95% Confidence Interval		
			Lower	Upper	p-value
CCI	0.765	0.0768	0.614	0.915	< .001

**Figure 3:**
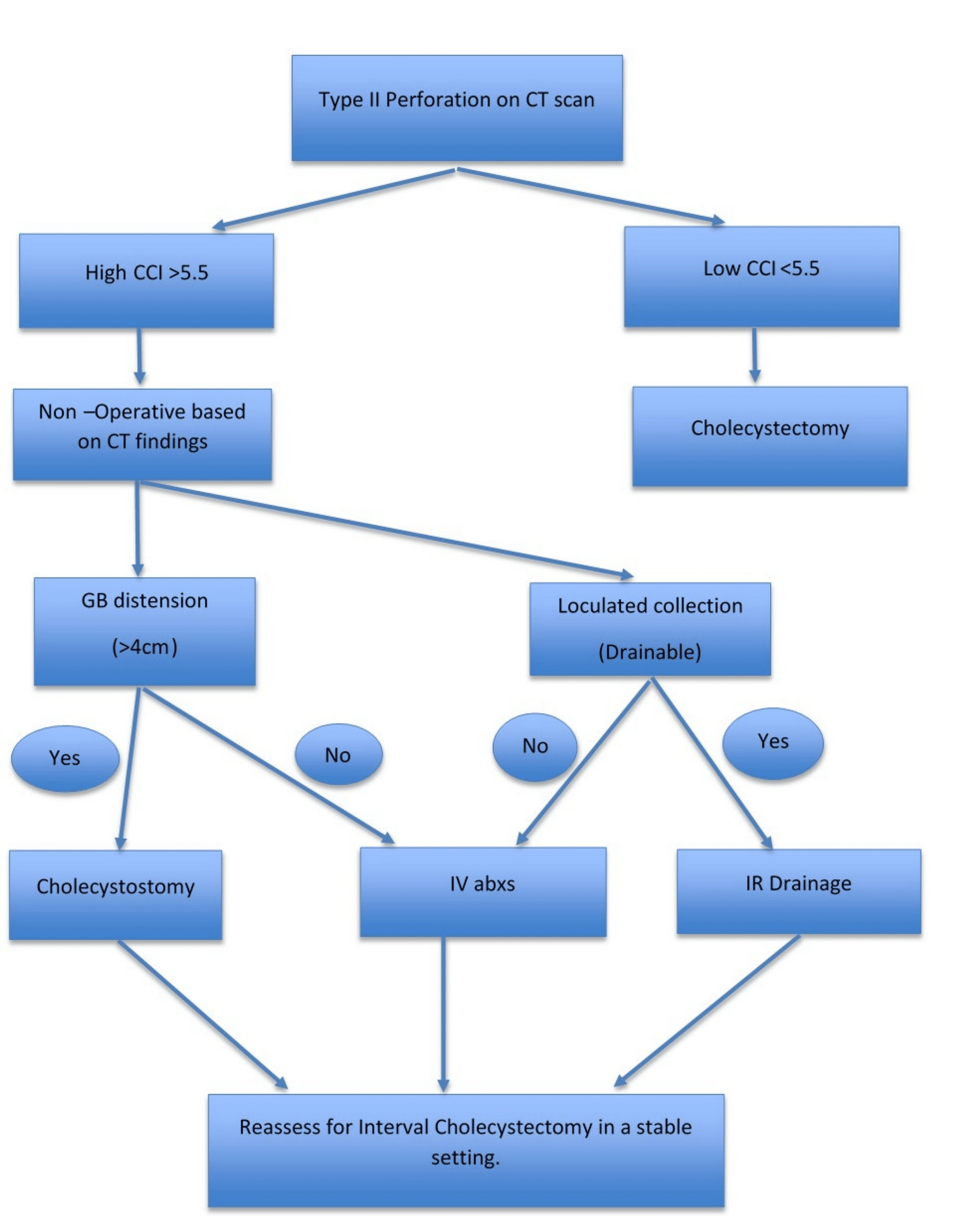
Algorithm of Management of Type II Perforation Based on CCI and CT Findings CCI: Charlson Comorbidity Index.

Based on the ROC analysis results and the observed associations between comorbidity burden and CT features with treatment selection, we developed a simplified management algorithm to guide individualized care (Figure 3). The algorithm incorporates the CCI and key radiological findings to support clinical decision-making in Type II GBP.

## Discussion

The management of localized GBP (Niemeier Type II) remains controversial, with no standardized guidelines across centers [5,7]. Type II accounted for nearly 80% of our cohort, consistent with the findings of Quiroga-Garza et al. [2], who identified localized perforation as the most frequent subtype across published series.

Multivariate analysis demonstrated that comorbidity burden, age, and radiological findings were key determinants of treatment choice. Patients with higher CCI scores and older age were more likely to receive conservative treatment, while surgery was largely reserved for fitter individuals with fewer comorbidities [3,7]. This reinforces the pivotal role of comorbidity in determining surgical fitness and clinician preference for conservative approaches [10]. Wani et al. similarly reported that frailty and comorbidity strongly influenced nonoperative selection [11], while Albisher et al. confirmed that higher CCI independently predicted worse outcomes in perforated gallbladder [3].

CT features were also important predictors in our series. Gallbladder distension was frequently associated with percutaneous cholecystostomy, consistent with interventional radiology guidance recommending decompression of an overdistended or septic gallbladder in high-risk patients [12,13]. Loculated pericholecystic collections tended to favor interventional drainage, paralleling previous reports where CT identified localized abscesses amenable to targeted drainage [14-16]. These findings highlight the central role of cross-sectional imaging in stratifying disease severity and directing the route of source control. Our ROC analysis further suggested that a CCI >5.5 effectively excluded patients from surgery. Although such a threshold has not been previously defined in the GBP literature, it aligns with wider evidence supporting comorbidity scores as predictors of outcome and guides to nonoperative selection [3].

We observed no significant differences in length of stay, mortality, or reintervention across the four management strategies, indicating that when treatment is individualized, conservative approaches can achieve outcomes comparable to surgery [17,18]. This mirrors the findings of Quiroga-Garza et al. [2], who reported similar short-term results across operative and nonoperative strategies. Singh et al. [19] likewise emphasized the importance of structured follow-up after nonoperative care due to the risk of recurrence. Collectively, these results support a selective rather than universal operative approach to Type II GBP.

Based on these findings, we propose a simplified algorithm incorporating CCI, age, and CT morphology to guide individualized management. Such a stratified approach may reduce mortality and avoid unnecessary intervention in high-risk patients unlikely to benefit from surgery [2,12,13,17,18]. Future multicenter validation is required, and defining the optimal balance between definitive surgery for low-risk patients and conservative strategies for high-risk cohorts remains an evolving field [20,21].

Limitations

This study has limitations. Its retrospective, single-center design is subject to selection bias, and management decisions were clinician-dependent rather than protocolized. In addition, small numbers in the surgical subgroup limited the statistical power for between-group comparisons. Finally, as this is a single-center retrospective study, the proposed algorithm should be validated in multicenter or resource-variable settings to assess its wider applicability.

## Conclusions

In this large single-center cohort, comorbidity burden, age, and CT morphology emerged as the principal determinants of management in Type II gallbladder perforation. When tailored to individual risk profiles, conservative strategies provided outcomes comparable to surgery in the short term. These findings underscore the importance of risk-stratified, imaging-guided decision making rather than a uniform operative approach. Future multicenter studies are required to validate these findings and to inform evidence-based guidelines for this complex condition.
